# Capturing of interdental areas with digital and conventional impression systems and their impact on automatic tooth segmentation in CAD software: an in vitro study

**DOI:** 10.1007/s00784-026-07069-2

**Published:** 2026-08-07

**Authors:** Katharina Klaus, Linus Vincent Hardt, Bernd Wöstmann, Sabine Ruf, Maximiliane Amelie Schlenz

**Affiliations:** 1 Department of Orthodontics, Dental Clinic of the Justus Liebig University Giessen, Schlangenzahl 14, 35392 Giessen, Germany; 2 Department of Prosthodontics, Dental Clinic of the Justus Liebig University Giessen, Giessen, Germany; 3 Private Dental Practice, Montabaur, Germany; 4 Department of Prosthodontics, Christian Albrecht University of Kiel, University Hospital Schleswig-Holstein, Campus Kiel, Kiel, Germany

**Keywords:** Intraoral scanners, Periodontally compromised dentition, Interdental areas, Digital impressions, Tooth segmentation

## Abstract

**Objectives:**

This in vitro study evaluated and compared the accuracy of digital versus conventional impression systems in reproducing interdental areas (IAs) of periodontally compromised dentitions (PCDs) and examined how impression quality influences automated tooth segmentation in CAD software.

**Materials and methods:**

A standardized PCD model was captured with one conventional polyvinylsiloxane impression (CVI), four wireless intra‑oral scanners (IOS) - Dexis IS 3800 W (3800‑wl), Medit i700w (i700‑wl), Primescan 2 (PRI2‑wl), Trios 5 (TRI5‑wl) - and a wired Primescan AC (PRI1‑w). Raw scan data, digitally mounted casts after scan‑repair, and digital casts after tooth segmentation in OnyxCeph were analyzed. IA reproduction accuracy and segmentation quality were statistically compared using the Median test and Chi‑squared test (*p* < 0.05).

**Results:**

All IOS reproduced IAs significantly more accurately than the CVI (*p* < 0.001). Among IOS, PRI2‑wl achieved > 80% correct IA reproduction, whereas 3800‑wl and TRI5‑wl fell short by up to 20%. Scan‑repair marginally improved IA capture for some IOS, while the conventional impression showed no change. Segmentation errors clustered in teeth with pronounced gingival recession; healthy gingiva yielded higher accuracy. Significant differences between raw and segmented data were found for TRI5‑wl (*p* = 0.027) and 3800‑wl (*p* = 0.044).

**Conclusions:**

Within the limits of this in vitro study, IOS outperform conventional impressions for IA reproduction in PCDs. Accuracy varies among IOS and is linked to raw‑scan quality. Automated segmentation is reliable for teeth with healthy gingiva but is challenged by periodontal defects.

**Clinical Relevance:**

Precise IA capture enhances orthodontic movement planning and streamlines CAD‑based workflows, reducing manual corrections and improving outcomes for patients with compromised periodontium.

## Introduction

Orthodontic treatment is no longer limited to children and adolescents, but has become increasingly common in adult patients [1, 2]. However, capturing the interdental areas (IAs) with digital and conventional impression systems remains particularly challenging in elderly patients due to age-related anatomical and physiological changes, as well as pathological tooth migration – often associated with attachment loss resulting from periodontitis, which affects up to 55.8% of patients [3, 4]. This migration leads to functional and aesthetic impairments, including elongation and flaring of the maxillary incisors, development of diastemas, anterior deep bite, and crowding of the mandibular incisors [1, 4]. Driven primarily by the desire to retain their natural teeth, improve esthetics, functional aspects, and overall well-being, interest in orthodontic tooth alignment was shown by 68% of adult patients with moderately to severely advanced periodontitis [5]. According to the 2021 Global Burden of Disease estimate, more than one billion people worldwide will be affected by severe periodontitis, with the highest prevalence in the age group of 50–64 years, regardless of global region [6].

In recent decades, there has been a noticeable trend toward increased demand for orthodontic treatment among adults, partly due to increased awareness of the link between oral health and overall well-being, and partly due to the availability of nearly invisible orthodontic devices such as clear aligners and fixed lingual braces [7, 8]. A recent survey exhibited that two-thirds of adult orthodontic patients prefer to use clear aligners [9]. Depending on the direction and extent of the desired tooth movements, as well as the magnitude of forces and anchorage requirements, aligners may represent the preferred treatment modality in patients with periodontally compromised dentitions (PCDs), offering the additional benefit of facilitating oral hygiene [10, 11].

The fabrication of orthodontic aligners requires highly accurate full-arch impressions or digital models to ensure correct segmentation and tooth movement [12, 13]. Conventional elastomeric impression materials, however, present specific challenges in dentitions affected by periodontal attachment loss. Pronounced undercuts and enlarged IAs, often referred to as open gingival embrasures or “black triangles”, facilitate the penetration of impression material into retentive areas. After setting of impression material, removal may be impeded by mechanical retention within these undercuts. Although elastomeric materials exhibit high elastic recovery and tensile strength, excessive removal forces may surpass their elastic limit, resulting in tearing or plastic deformation. Consequently, accurate capturing of IAs may be compromised in PCDs.

As intraoral scanning increasingly replaces conventional impression techniques in clinical practice, *Schlenz et al.* evaluated the accuracy of digital impression systems in capturing IAs in PCDs [14, 15]. Although intraoral scanners (IOS) demonstrated superior performance compared to conventional impression methods, their accuracy in capturing IAs remained limited.

In recent years, the IOS market has undergone substantial technological advancement, including improvements in trueness and precision, increased scan depths, and the introduction of wireless handpieces to enhance clinical flexibility. In September 2024, Dentsply Sirona introduced a cloud-based IOS architecture that relocates data processing and storage to remote servers [16]. While this approach reduces local hardware requirements and facilitates mobile deployment, it also raises concerns regarding data transfer speed, latency, and potential effects on system accuracy, particularly in regions with limited or unstable internet connectivity [17].

Given the continuous evolution of both scanning hardware and software, it may be hypothesized that the accuracy of capturing interdental areas has improved since the investigations published in 2019 and 2020 [14, 15]. To the best of the author’s knowledge, no study to date has evaluated the performance of the current generation of wireless and cloud-based IOS systems with respect to the capturing and reproduction of IAs in PCDs. Artificial intelligence (AI)-based tools are increasingly integrated into computer-aided design (CAD) software to facilitate automated post-processing steps, such as removal of irrelevant soft-tissue areas (e.g. lips and tongue), surface reconstruction (“scan repair”), and tooth segmentation. However, in clinical practice, incomplete or inaccurate capture of IAs frequently results in segmentation errors, necessitating time-consuming manual correction by the dentist [18]. This observation suggests that inadequate capturing of IAs remains a relevant limitation for current algorithms.

Reliable capture of IAs directly impacts the precision, efficiency, and predictability of orthodontic aligner treatment in patients with PCD. Therefore, the aim of this in vitro study was to provide an updated comparison of conventional and contemporary digital impression systems, including wireless and cloud-based IOS, with regard to their ability to capture IAs and their influence on automated tooth segmentation in CAD software (OnyxCeph^3TM^ Image Instruments, Chemnitz, Germany), using an established model of a PCD.

The following null hypotheses were tested:

- there is no difference among the impression systems in capturing of IAs;
- there is no difference in reproduction of IAs among raw data of impression systems, digitally mounted casts after scan repair in OnyxCeph^3TM^, and digital casts after tooth segmentation; and
- there is no difference in tooth segmentation outcomes among casts digitally processed on the basis of the different impression systems.

## Materials & methods

**Fig. 1:**
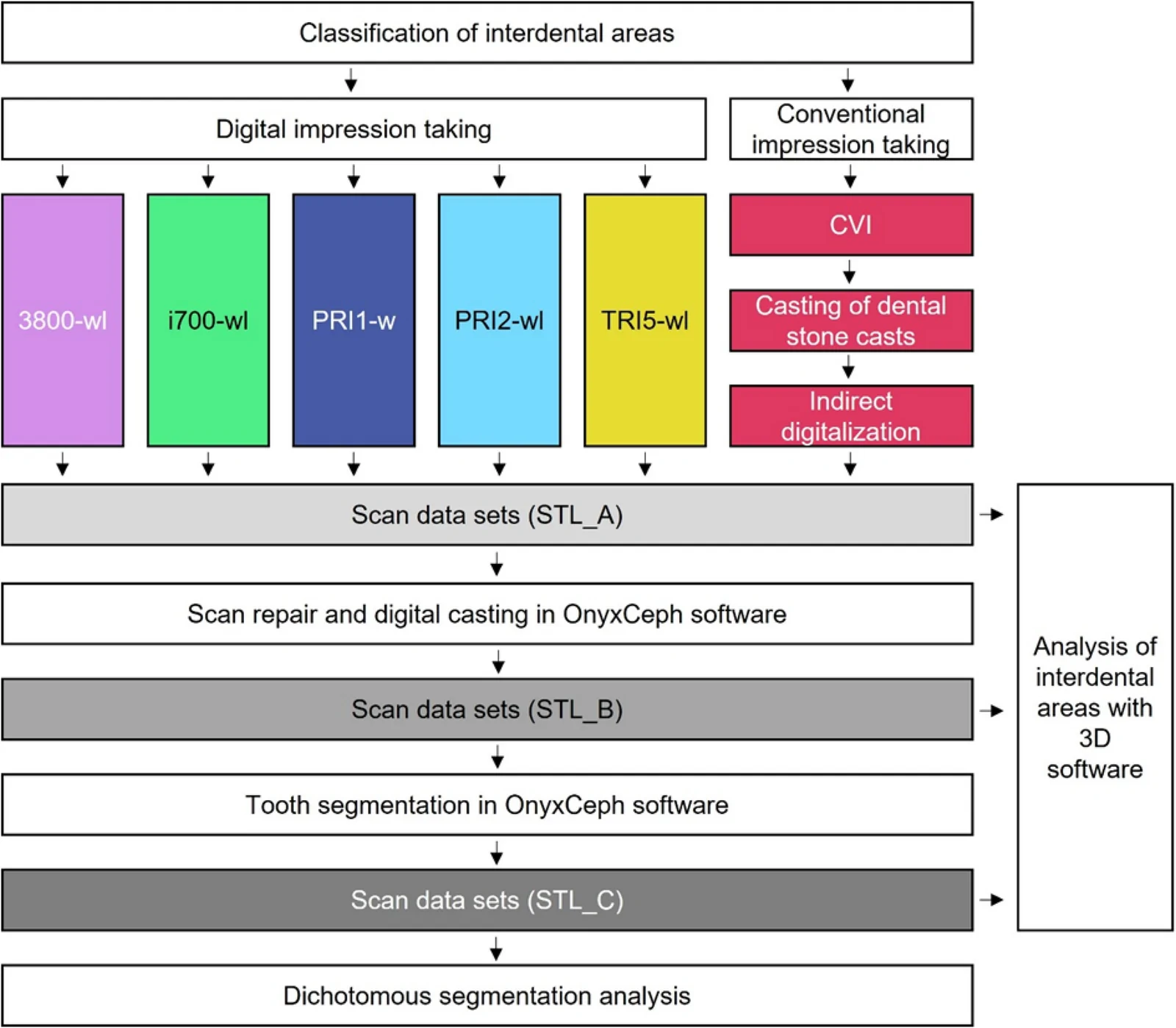
Flow scheme of the experimental procedure (3800-wl = Dexis IS 3800 W wireless; i700-wl = Medit i700w wireless; PRI1-w = Primescan AC wired; PRI2-wl = Primescan 2 wireless; TRI5-wl = Trios 5 wireless; CVI = conventional impression; STL_A = raw data of impression systems; STL_B = digitally mounted casts after scan repair in OnyxCeph; STL_C = digital casts after tooth segmentation)

The present study was conducted at the Department of Prosthodontics and the Department of Orthodontics of the Justus Liebig University Giessen (Germany) between March and October 2024. Due to the in vitro approach, no ethical approval was needed. The methodology used in this in vitro study is based on the approach first described by *Schlenz et al.* [14]. A flow scheme of the experimental procedure is displayed in Fig. 1. To ensure standardized testing conditions, the same dentist (L.H.), who was experienced with all the impression systems investigated and trained in the CAD and 3D analysis software, performed the impression taking and measurements.

### Test model

**Fig. 2:**
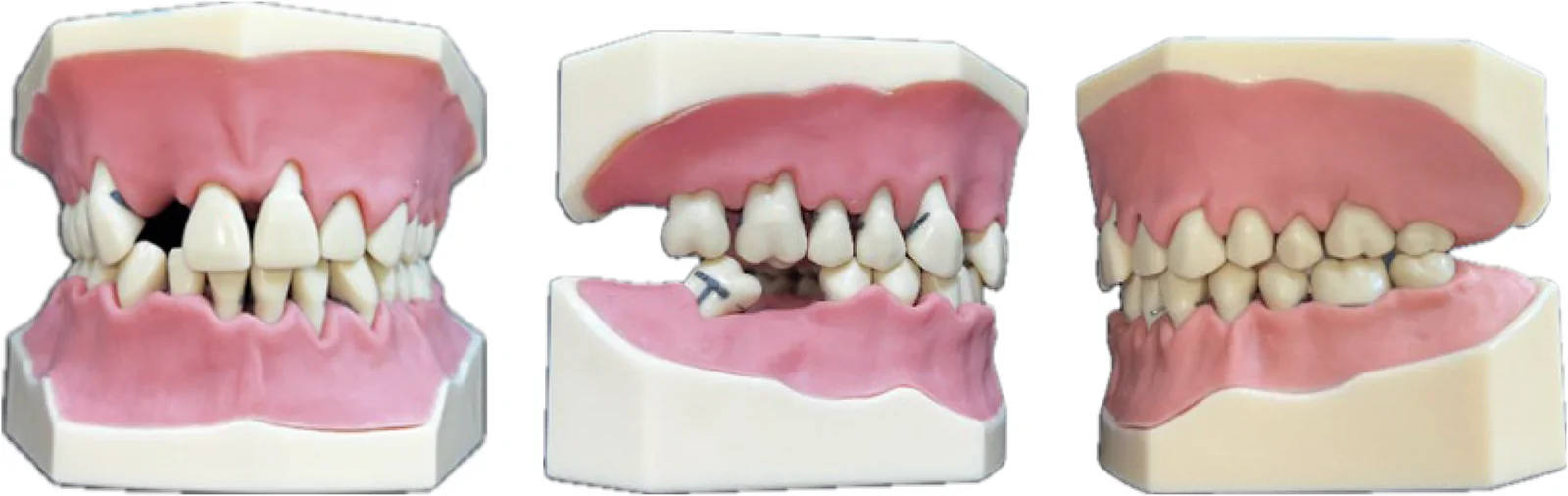
Test model (A-PB, Frasaco) with periodontally compromised dentition

**Fig. 3:**
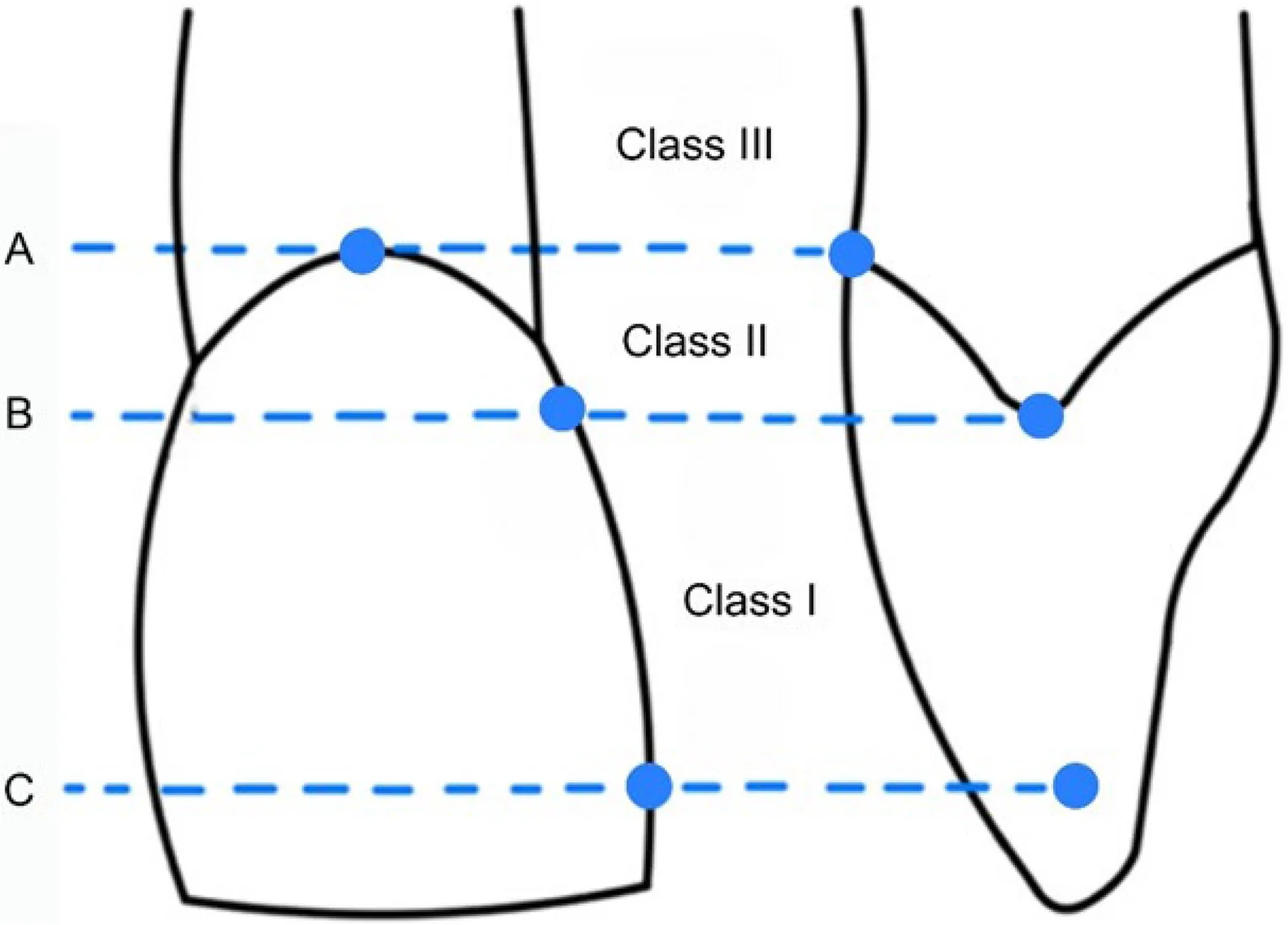
Schematic drawing of the classification system according to *Nordland and Tarnow* [19] with (**a**) facial cemento-enamel junction (CEJ), (**b**) interproximal CEJ, (**c**) interproximal contact

A standardized test model representing the upper and lower jaws of a permanent dentition with advanced periodontitis affecting both the alveolar bone and the gingiva (A-PB, Frasaco, Tettnang, Germany) was used. In this model, teeth 12 and 46 (FDI classification) were missing, resulting in one edentulous space and eleven remaining IAs per jaw (Fig. 2). Each IA was initially classified according to the *Nordland and Tarnow* criteria [19] (Fig. 3; Tables 1 and 2). Impressions were taken using a phantom head to simulate a more clinically relevant study setup.

**Table 1:** Classification of interdental areas (IA) according to *Nordland and Tarnow* [19]

Classification	Description
Normal	The interdental papilla fills the embrasure space to the apical extent of the interdental contact point / area.
Class I	The tip of the interdental papilla is located between the interdental contact point and the most coronal extent of the cemento-enamel junction (CEJ) (space present but interproximal CEJ is not visible).
Class II	The tip of the interdental papilla is located at or apical to the interdental CEJ, but coronal to the apical extent of the facial CEJ (interproximal CEJ is visible)
Class III	The tip of the interdental papilla is located even or apical to the facial CEJ.

**Table 2:** Classification for each interdental area (IA) of the test model according to *Nordland and Tarnow* [19]

						upper jaw					
IA	17/16	16/15	15/14	14/13	11/21	21/22	22/23	23/24	24/25	25/26	26/27
Class	II	II	III	I	II	I	I	I	I	I	normal
						lower jaw					
IA	45/44	44/43	43/42	42/41	41/31	31/32	32/33	33/34	34/35	35/36	36/37
Class	I	II	II	II	III	II	II	II	II	II	I

### Digital and conventional impression taking

Under laboratory conditions (room temperature: 23 °C ± 1 °C, humidity: 50% ± 10%), the upper and lower jaws were digitally scanned twelve times each with five intraoral scanners (IOS):

- Dexis IS 3800 W wireless (‘3800-wl’, EH Germany, Herzogenrath, Germany; version 1.0.9.832).
- Medit i700w wireless (‘i700-wl’, Medit, Seoul, South Korea; version 3.2.1).
- Primescan AC wired (‘PRI1-w’, Dentsply Sirona, Bensheim, Germany; version 5.2.8).
- Primescan 2 wireless and cloud-based (‘PRI2-wl’, Dentsply Sirona; version 1.1.1).
- Trios 5 wireless (‘TRI5-wl’, 3Shape, Copenhagen, Denmark; version 22.1.1).

If provided by the manufacturer, the IOS were calibrated prior to use according to the manufacturer’s instructions. A standardized scanning protocol was followed, beginning with the occlusal surfaces, continuing with the palatal/lingual surfaces, and ending with the buccal surfaces [20]. To ensure adequate capture of the IAs, the scanner tips were slightly tilted at various angles until no further improvement in IA visibility was observed in the scanning software. A constant distance of 10 ± 5 mm was maintained throughout the scanning process. The datasets from all IOS were saved and exported as Standard Tessellation Language (STL) files for further analysis (STL_A: raw data of impression systems).

Conventional impressions (CVIs) were taken with polyvinylsiloxane (EXA’lence putty, batch no. 2310061, and EXA’lence light body, batch no. 2110041; GC, Tokyo, Japan) using the one-step putty-wash technique. Twelve impressions per jaw were taken with a full-arch metal tray (Ehricke stainless steel perforated BO-2/BU-2, Omnident, Rodgau, Germany). After storage for at least two hours, the impressions were casted in type IV dental stone (Fujirock EP, batch no. 2311303; GC). The plaster models obtained from the CVI were then digitized using an industrial scanner (ATOS Core 135, Zeiss IQS, Oberkochen, Germany). STL files were exported for further analysis (STL_A: raw data of impression systems).

### Dataset analysis with CAD software OnyxCeph^3TM^

**Fig. 4:**
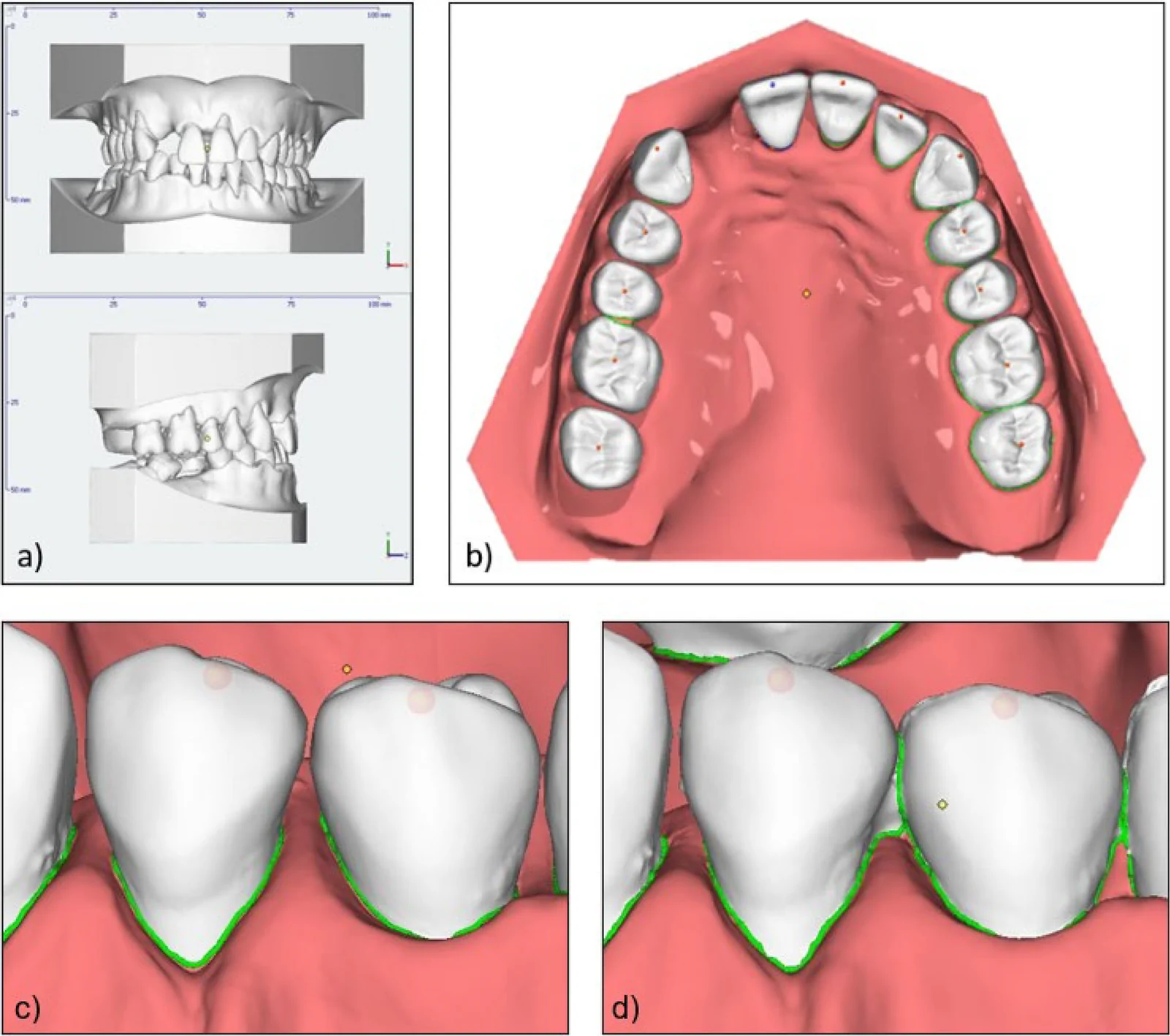
(**a**) Digitally aligned and mounted digital model in OnyxCeph^3TM^; (**b**) Segmentation of digital models in OnyxCeph^3TM^. Red dots = manually marked centers of the teeth. Green lines = margins of the teeth identified by the underlying algorithm of OnyxCeph^3TM^; (**c**) Correct segmentation of IA between teeth 34 and 35; (**d**) Incorrect segmentation of IA with need for manual adjustment of the margin at tooth 34.

STL files from both digital and conventional impression systems (STL_A: raw data of impression systems) were imported into the CAD software OnyxCeph^3TM^ (Image Instruments, software version 3.2.223 (608)) and processed according to the manufacturer’s instructions. To create a digital model, the scans were aligned using the raphe mediana and the occlusal plane as reference structures. A built-in scan repair function checked the files for potential errors (such as invalid elements, islands, holes, bridges, incorrect element orientation or overlap) and corrected them. This process was repeated until only minimal (non-repairable) errors remained. Next, the edges of the intraoral scans were trimmed in the model alignment module and the scans were mounted using a prefabricated base (Fig. 4a). The newly created STL files were then exported for further analysis (STL_B: digitally mounted casts after scan repair in OnyxCeph^3TM^).

Based on these STL files (STL_B: digitally mounted casts after scan repair), automatic tooth segmentation was then performed using the segmentation module of OnyxCeph^3TM^. The appropriate teeth were selected and the software calculated the tooth margins based on automated suggestions derived from the underlying algorithms (Fig. 4b). Following the manufacturer’s instructions, the exact center of the occlusal surface was manually marked for molars and premolars, the center of the incisal edge for incisors, and the cusp tip for canines. To evaluate tooth segmentation, a dichotomous yes/no decision was made to determine whether the teeth were correctly segmented by the algorithm (Fig. 4c). If manual corrections were required, the tooth was considered “incorrect” and the margins were adjusted (Fig. 4d). Subsequently, the segmented models were also exported as STL files for subsequent analysis of the IAs (STL_C: digital casts after tooth segmentation).

### Measurement of interdental areas (IAs)

**Fig. 5:**
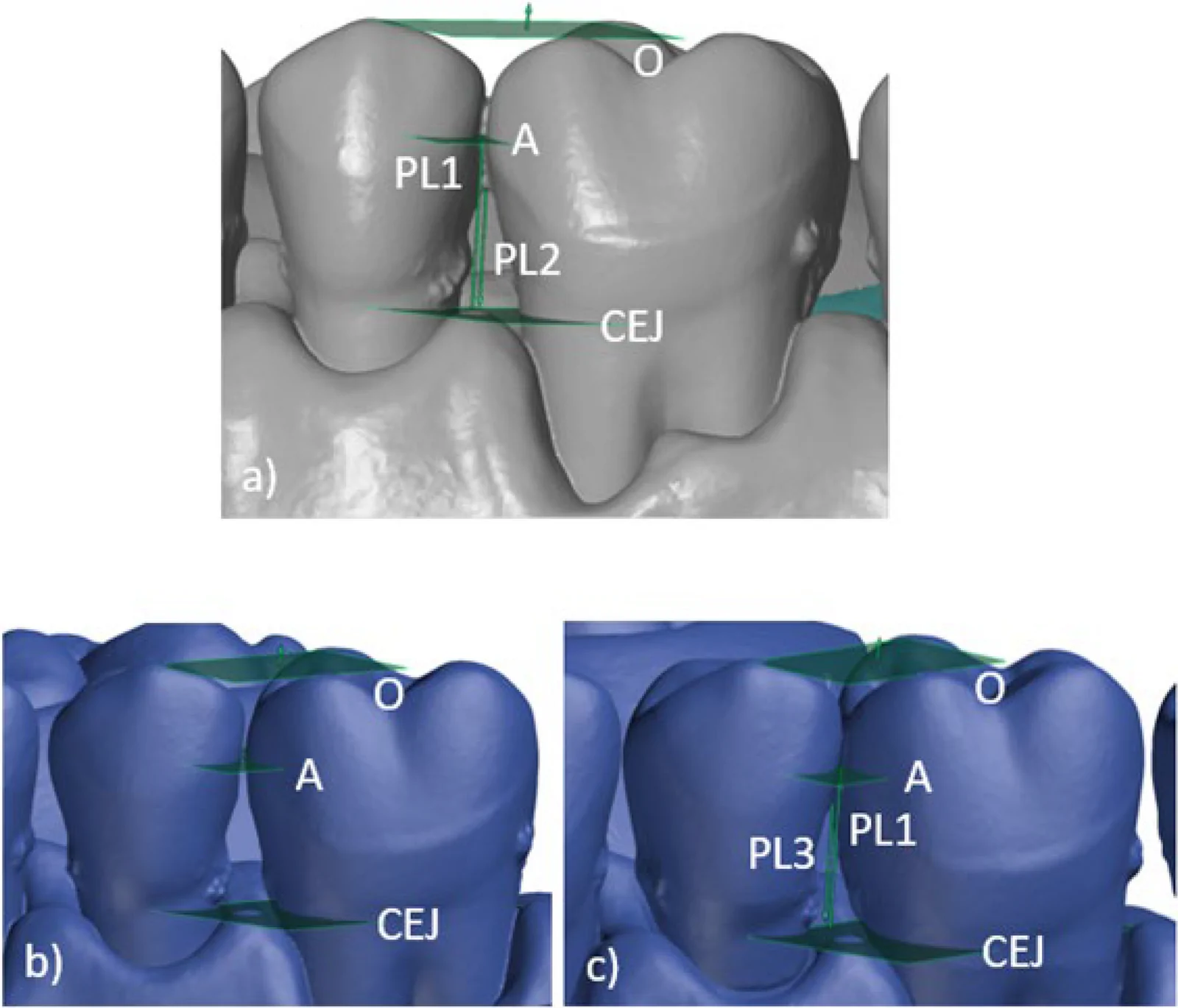
Example for measurement of interdental areas. O = occlusal plane, A = approximal plane, CEJ = cemento-enamel junction, PL = perpendicular length. (**a**) STL_A representing the raw data, IA = PL2/PL1 × 100. (**b**) STL_B after scan repair and digital casting. (**c**) STL_C after digital tooth segmentation, IA = PL3/PL1 × 100

Finally, all STL files (STL_A: raw data of impression systems, STL_B: digitally mounted casts after scan repair, STL_C: casts after tooth segmentation) were imported into the Zeiss Inspect Optical 3D software (Zeiss IQS, Oberkochen, Germany; software version 2023.3.0.969) to measure the IAs. Therefore, three reference planes were created on the STL_A for each individual IA: the occlusal plane (O), the cementoenamel junction (CEJ), and the approximal plane (A), which was assumed to be 3.0 mm apical to and parallel to the occlusal plane for standardization purposes. The IAs were measured using two perpendicular lengths (PL): PL1, between A and the CEJ, and PL2, between the CEJ and the most coronal point of the IA. The following formula was then used to calculate a percentage for each IA: (Fig. 5a).

*IA=(PL2(mm))/(PL1(mm))×100*

In the next step, STL_B and STL_C were superimposed on STL_A using a best-fit superimposition method. To evaluate the IAs after scan repair (STL_B), no additional perpendicular line had to be created because there were either no proximal contacts in these models, resulting in maximum IA display (100%, Figs. 4c and 5b) or the IA was completely closed, resulting in minimum IA display (0%, Fig. 4d). To measure the IAs after tooth segmentation, a third perpendicular length (PL3) was created between the CEJ and the most coronal point of the interproximal area, which was assumed by the orthodontic evaluation program after segmentation. The following formula was then used to analyze the IA = (PL3 (mm)) / (PL1 (mm)) × 100 (Fig. 5c).

### Statistical analysis

Statistical analysis was performed using SPSS statistics (version 28, IBM Corp., Armonk, NY, USA). For analysis of IA measurements, the median test was applied, since the data revealed several 0 and 100 values with partial statistical outliers. Finally, p-values were corrected using the Bonferroni-Holm method due to the risk of alpha-error accumulation. Tooth segmentation was analyzed using Chi-square tests. The level of significance was set at p-value < 0.05. To assess reliability, twenty randomly selected STL files were remeasured by the same investigator (L.H.) after a minimum of four weeks, and the intraclass correlation coefficient was calculated. Intra-examiner reliability was found to be excellent (ICC = 0.982 to 1.000).

## Results

**Fig. 6:**
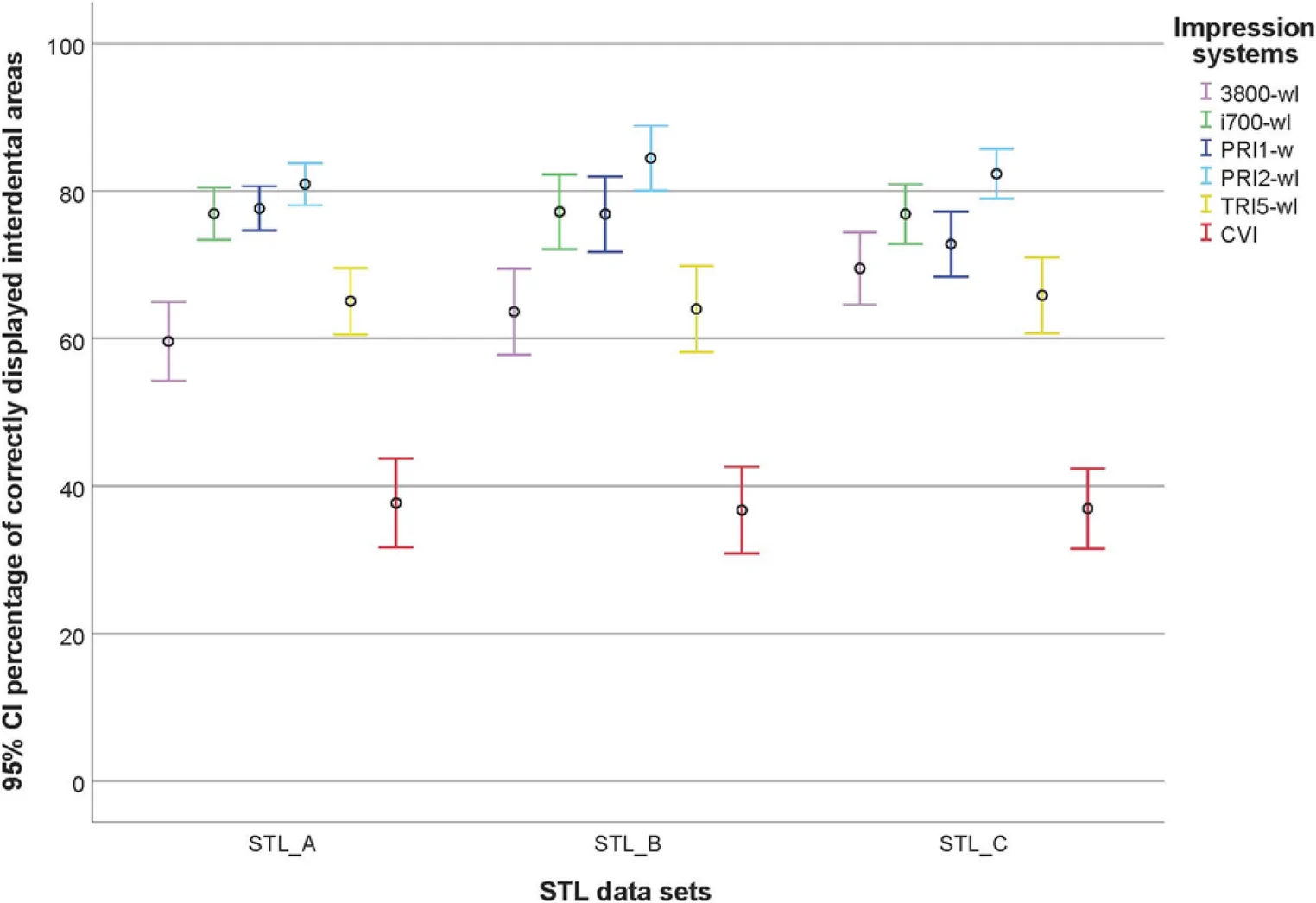
The 95% confidence interval of the displayed interdental areas for the six different impression systems distributed to the three STL data set groups at the different steps of digital processing (STL_A = raw data of impression systems; STL_B = digitally mounted casts after scan repair in OnyxCeph^3TM^; STL_C = digital casts after tooth segmentation)

In total, 4,752 IAs were analyzed. Across all stages of data processing, the digital impression systems captured IAs significantly more accurately than the conventional impressions (*p* < 0.001). Although differences between the digital systems were apparent, they did not reach statistical significance. Overall, PRI2-wl consequently achieved the highest performance, correctly reproducing over 80% of IAs, whereas 3800-wl and TRI5-wl captured up to 20% less IAs across all process steps (Fig. 6). Consequently, the first null hypothesis (a) must be rejected.

While the reproduction of IAs in conventional impressions remained unaffected by subsequent digital processing, a trend was observed in all digital systems towards equal or slightly improved IA capture following digital scan repair (STL_B), accompanied by increased variability compared to the raw scan data (STL_A). After tooth segmentation (STL_C), IA reproduction remained stable for PRI2-wl, i700-wl and TRI5-wl, whereas the 3800-wl system exceeded its initial raw scan data values and PRI1-w showed a slight decrease (Fig. 6). Statistically significant differences were detected between the raw data (STL_A) and digital casts after tooth segmentation (STL_C) for TRI5-wl (*p* = 0.027) and 3800-wl (*p* = 0.044). Consequently, the second null hypothesis (b) must be rejected.

**Fig. 7:**
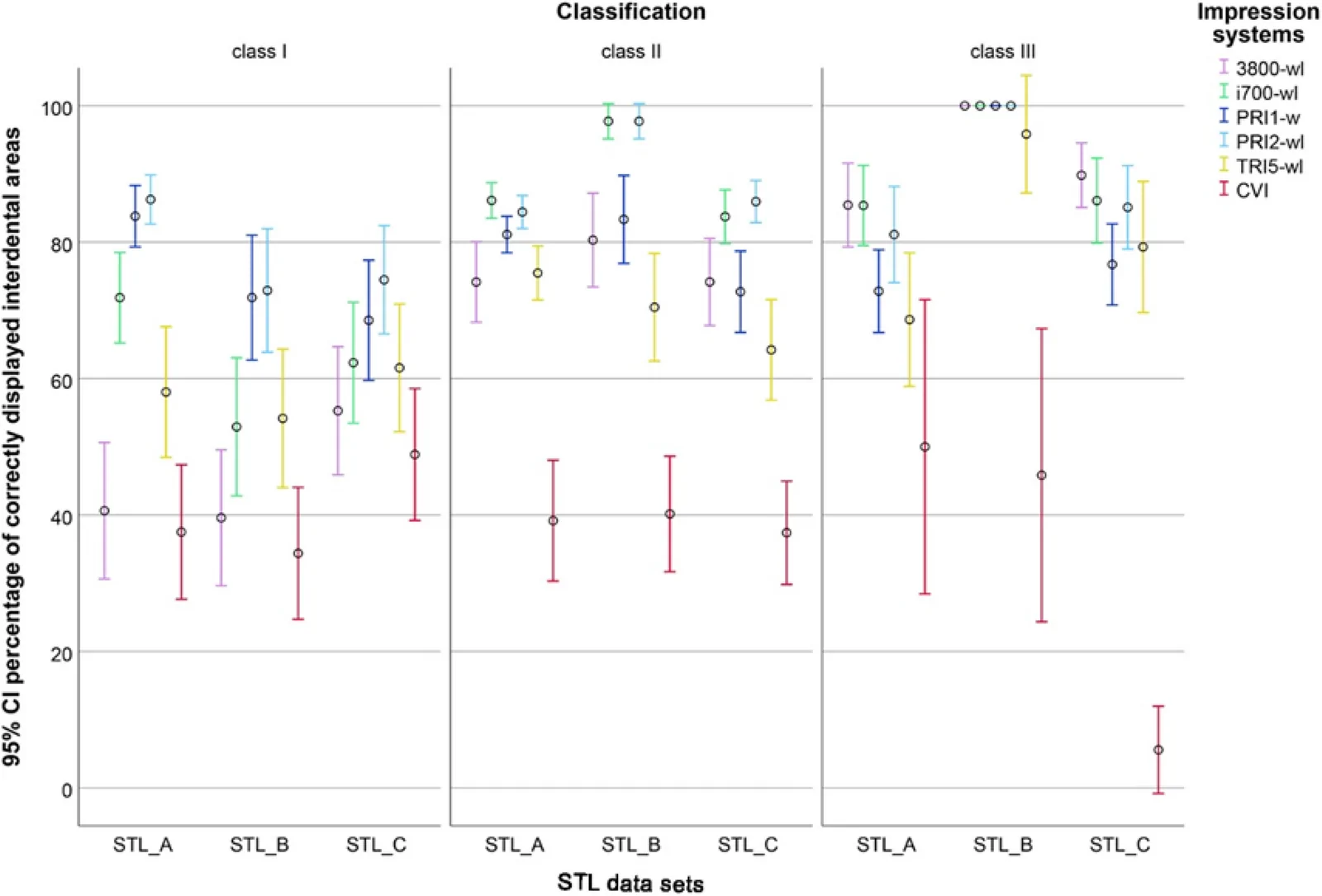
The 95% confidence interval of the displayed interdental areas for the six different impression systems classified by *Nordland and Tarnow* [19] distributed to the three STL data set groups at the different steps of digital processing (STL_A = raw data of impression systems; STL_B = digitally mounted casts after scan repair in OnyxCeph^3TM^; STL_C = digital casts after tooth segmentation)

**Fig. 8:**
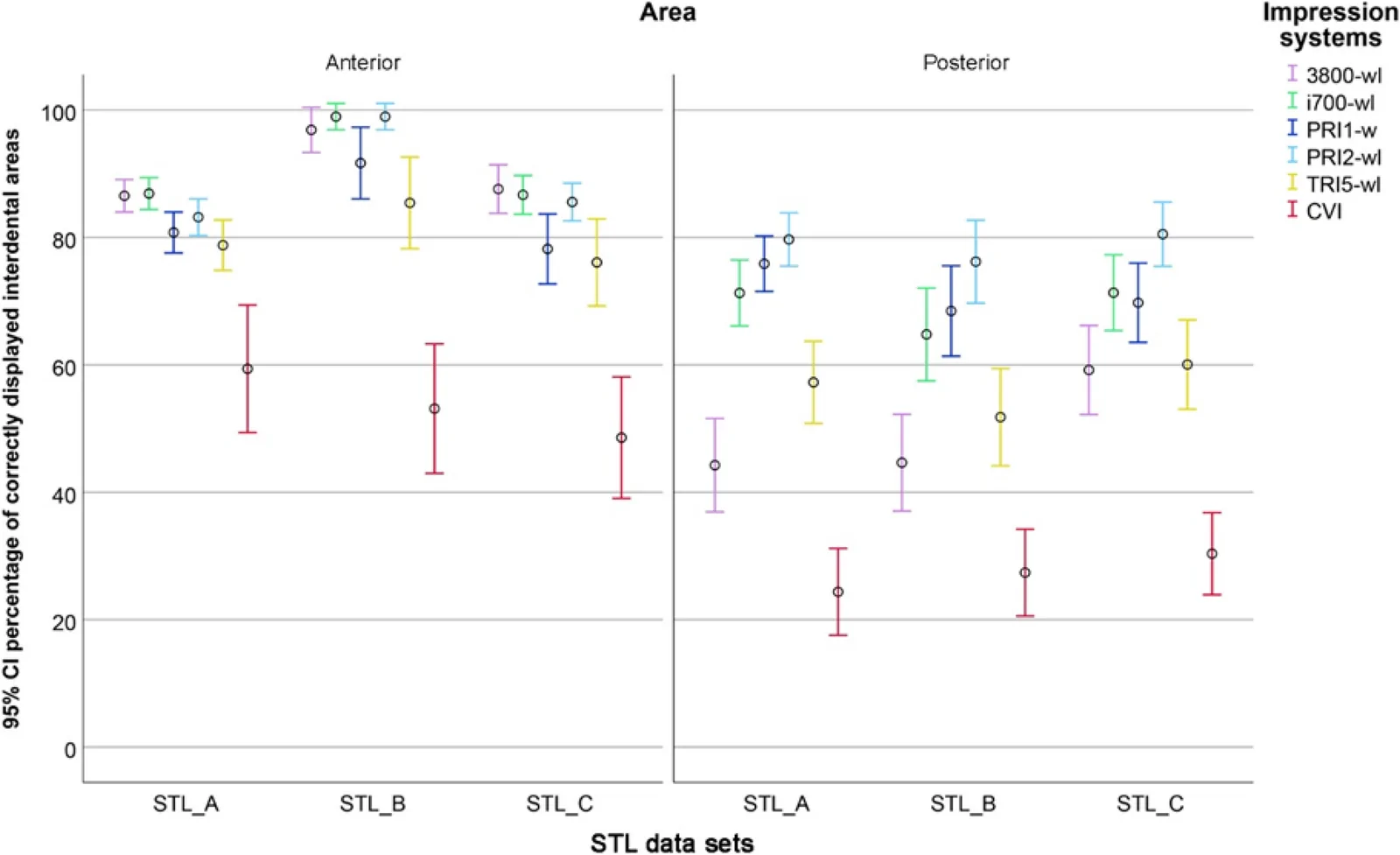
The 95% confidence interval of the displayed interdental areas for the six different impression systems displayed separately for the anterior and posterior area of the jaws distributed to the three STL data set groups at the different steps of digital processing (STL_A = raw data of impression systems; STL_B = digitally mounted casts after scan repair in OnyxCeph^3TM^; STL_C = digital casts after tooth segmentation)

Regarding the classification of IAs according to *Nordland and Tarnow* [19], the digital systems achieved higher accuracy with increasing IA width (Fig. 7; Table 3). All systems reproduced anterior IAs more reliably than posterior ones, with the difference between the digital systems and the CVIs being more pronounced in the anterior region (Fig. 8).

**Table 3:** Descriptive statistics and results of the median test corrected by the Bonferroni-Holm method. Statistically significant differences are highlighted with an asterisk

Region	Classifi-cation	Impression Technique	Descriptive Statistics			p-values (corrected by Bonferroni-Holm)				
			25th percentile	median	75th percentile	TRI5-wl	3800-wl	PRI1-w	i700-wl	PRI2-wl
anterior region	Class I	CVI	100.00	100.00	100.00	1	1	1	1	1
		TRI5-wl	100.00	100.00	100.00	-	1	1	1	1
		3800-wl	100.00	100.00	100.00	-	-	1	1	1
		PRI1-w	100.00	100.00	100.00	-	-	-	1	1
		i700-wl	100.00	100.00	100.00	-	-	-	-	1
		PRI2-wl	100.00	100.00	100.00	-	-	-	-	-
	Class II	CVI	0.00	0.00	100.00	1	1	1	1	1
		TRI5-wl	67.93	84.57	100.00	-	0.121	0.715	0.110	1
		3800-wl	79.81	100.00	100.00	-	-	< 0.001*	0.930	0.039*
		PRI1-w	71.19	79.84	100.00	-	-	-	< 0.001*	0.252
		i700-wl	80.22	90.50	100.00	-	-	-	-	0.036*
		PRI2-wl	76.22	86.18	100.00	-	-	-	-	-
	Class III	CVI	0.00	0.00	87.90	1	1	1	1	1
		TRI5-wl	48.90	70.19	100.00	-	< 0.001*	< 0.001*	< 0.001*	< 0.001*
		3800-wl	74.69	79.55	100.00	-	-	0.01*	1	0.918
		PRI1-w	61.57	68.19	100.00	-	-	-	< 0.001*	1
		i700-wl	70.32	79.56	100.00	-	-	-	-	0.816
		PRI2-wl	66.58	74.06	100.00	-	-	-	-	-
posterior region	Class I	CVI	0.00	0.00	0.00	< 0.001*	1	< 0.001*	< 0.001*	< 0.001*
		TRI5-wl	0.00	0.00	100.00	-	< 0.001*	0.048*	0.08	0.056
		3800-wl	0.00	0.00	70.83	-	-	< 0.001*	< 0.001*	< 0.001*
		PRI1-w	0.75	100.00	100.00	-	-	-	0.138	0.739
		i700-wl	0.00	57.06	100.00	-	-	-	-	0.04*
		PRI2-wl	60.93	100.00	100.00	-	-	-	-	-
	Class II	CVI	0.00	0.00	100.00	< 0.001*	< 0.001*	< 0.001*	< 0.001*	< 0.001*
		TRI5-wl	3.20	100.00	100.00	-	1	1	1	1
		3800-wl	0.00	100.00	100.00	-	-	1	1	1
		PRI1-w	71.69	100.00	100.00	-	-	-	1	1
		i700-wl	91.15	100.00	100.00	-	-	-	-	1
		PRI2-wl	84.92	100.00	100.00	-	-	-	-	-
	Class III	CVI	0.00	0.00	100.00	1	1	1	1	1
		TRI5-wl	89.72	100.00	100.00	-	1	1	1	1
		3800-wl	100.00	100.00	100.00	-	-	1	1	1
		PRI1-w	83.11	96.18	100.00	-	-	-	1	1
		i700-wl	100.00	100.00	100.00	-	-	-	-	1
		PRI2-wl	100.00	100.00	100.00	-	-	-	-	-

With respect to tooth segmentation performed by the CAD software, statistically significant differences between the initial impression systems were observed for the majority of teeth (Table 4). Therefore, the third null hypothesis (c) must be rejected. Overall, there was good agreement among the digital impression systems for both correctly or incorrectly segmented teeth, with segmentation errors occurring predominantly in teeth exhibiting pronounced periodontal recessions.

**Table 4:** Percentage of correct tooth segmentation in the CAD software for the different impression techniques and teeth

							upper jaw						
Impression technique	17	16	15	14	13	11	21	22	23	24	25	26	27
CVI	83.3	16.6	8.3	8.3	0.0	0.0	0.0	8.3	33.3	41.6	16.6	25.0	25.0
TRI5-wl	100.0	0.0	75.0	0.0	0.0	0.0	0.0	100.0	100.0	100.0	100.0	75.0	100.0
3800-wl	100.0	0.0	41.6	0.0	0.0	0.0	0.0	100.0	100.0	100.0	100.0	100.0	100.0
PRI1-w	100.0	0.0	91.6	0.0	0.0	0.0	0.0	100.0	100.0	100.0	100.0	100.0	100.0
i700-wl	100.0	0.0	100.0	0.0	0.0	0.0	0.0	100.0	100.0	100.0	100.0	100.0	100.0
PRI2-wl	100.0	0.0	100.0	0.0	0.0	0.0	0.0	100.0	100.0	100.0	100.0	100.0	100.0
*p*-value	< 0.001	0.068	< 0.001	0.407	1	1	1	< 0.001	< 0.001	< 0.001	< 0.001	< 0.001	< 0.001
							lower jaw						
Impression technique	37	36	35	34	33	32	31	41	42	43	44	45	47
CVI	66.6	16.6	33.3	41.6	0.0	0.0	0.0	0.0	8.3	0.0	0.0	41.6	16.6
TRI5-wl	100.0	0.0	100.0	100.0	0.0	0.0	0.0	0.0	100.0	100.0	100.0	100.0	100.0
3800-wl	91.6	66.6	75.0	75.0	0.0	0.0	0.0	0.0	100.0	100.0	91.6	100.0	100.0
PRI1-w	100.0	33.3	100.0	100.0	0.0	0.0	0.0	0.0	75.0	100.0	100.0	100.0	100.0
i700-wl	100.0	75.0	100.0	100.0	0.0	0.0	0.0	0.0	100.0	100.0	100.0	100.0	100.0
PRI2-wl	100.0	58.3	100.0	100.0	0.0	0.0	0.0	0.0	100.0	100.0	100.0	100.0	100.0
*p*-value	0.005	< 0.001	< 0.001	< 0.001	1	1	1	1	< 0.001	< 0.001	< 0.001	< 0.001	< 0.001

## Discussion

Over the past decade, accumulating evidence has demonstrated that IOS are at least equivalent respectively superior to CVI in terms of accuracy and efficiency for orthodontic appliances [21, 22]. However, patients with PCD represent a unique challenge in orthodontics due to their elevated demands for diagnostic precision, therapeutic control and oral hygiene management.

For capturing IAs in PCDs, the present investigation confirmed the superiority of IOS compared to CVIs, which is in concordance with previous results [14, 15]. Especially the analysis of the further digital processing steps underlined the importance of high-quality input into the workflow (Fig. 6). Although only minor changes reached statistical significance, some IOS showed a tendency towards increased IA reproduction during subsequent digital processing, whereas the digitized CVI data remained unchanged.

According to the IA classification of *Nordland and Tarnow* [19], the increasing range of classification reflects an IA increase in width as well as depth. This revealed some interesting findings: First, class I IAs, which are closest to the normal physiologic IA, obtained the largest heterogeneity within the IOS. During further processing, most IOS presented a considerable decrease in accuracy, which was partly corrected in the last processing step, but still did not reach the original accuracy values of raw scan data in i700-wl, PRI1-w and PRI2-wl. Conversely, 3800-wl and TRI5-wl, which showed lower initial values in raw scan data, improved during subsequent post-processing. Second, class II IAs reflect the gap between IOS and CVI most obviously. While all IOS provide acceptably accurate raw scan data, CVI displayed about 50% less IAs correctly. During further digital processing, PRI2-wl and i700-wl performed considerably better than the other IOS which were suspected to an increased scattering. Third, increased scattering of all techniques was also noticed in class III IAs, which are the widest and deepest ones. While PRI2-wl performed well in all IA classifications, 3800-wl and i700-wl showed a considerably higher accuracy with increasing IA width and depths. This effect may be attributable to the underlying imaging principles of the different scanners. Systems based on active triangulation, in particular, may achieve higher accuracy in wider IAs, as the increased spacing facilitates more precise trigonometric surface reconstruction. Interestingly, PRI2-wl achieved constantly better results compared to the PRI1-w, despite the fact that they share similar hardware components. Accordingly, the developments in software and cloud-based outsourcing of computing power seems to influence the performance of the IOS.

Based on the former investigations of IOS performance in PCDs [14, 15], improvements in scanning accuracy were seen. Compared to the predecessor model CS3600, the 3800-wl showed slight improvements in IA display [15]. More obvious advancements were seen in the TRI5-wl compared to its predecessor TRIOS 3 [14, 15]. The PRI1-w was also tested previously and performed much better in the present investigation [15] – this could either be influenced by further advancement of the scanning software or by the larger variability and heterogeneity of the in vivo patient sample in the former study compared to the actual in vitro approach [15]. The latter seems to be relevant to a certain extent because even the CVI obtained better results for the anterior segments in the current study compared to 2020.

Irrespective of classification, a higher proportion of IAs was visualized in the anterior region compared to the posterior region. This is in line with previous results [14, 15]. Either in the current in vitro setting using a phantom head or even more in vivo, the anterior area offers superior accessibility, as soft tissues such as the tongue, lips, and cheeks can be retracted more easily. In addition, greater angulation of the scanner handpiece is feasible in the anterior region, facilitating the acquisition of IAs. The anatomical characteristics of anterior teeth, which are typically narrower compared to posterior ones, often result in more delicate contact points with smaller proximal surfaces. Moreover, anterior IAs are generally less deep than posterior IAs due to the oro-vestibular extension of the teeth and the alveolar process. By contrast, spatial conditions in the posterior region are considerably more restricted and angulation of the scanner handpiece - particularly in the buccal area - is only possible to a limited extent. As the angle of the emitted light from optical systems is constrained by handpiece angulation, undercuts are more difficult, or in some cases impossible, to capture within the focal plane.

In terms of tooth segmentation, significant differences were seen especially between CVI and the digital systems. Nevertheless, limitations in tooth segmentation capacity even for digital impressions were observed in teeth 16, 13, 11, 21, 33, 32, 31 and 41, which all presented prominent recessions either buccally or lingually. Some heterogeneity for IOS were visible in teeth 15 and 36 for less obvious reasons. All in all, tooth segmentation using the underlying algorithms in the CAD software are performed better in STL datasets based on digital impression systems. Actually, there is a large increase in development and validation of automated AI-driven clinical workflows as digital tooth segmentation [23–28]. A recent systematic review found that AI-driven tooth segmentation of IOS data in general is superior to human performance in terms of speed and efficiency, but with varying accuracy depending on the type of teeth as well as the jaws [29]. However, further need for improvements of gingival boundary segmentations has been claimed [29], which is in concordance with the present results. Some studies compared the tooth segmentation performance of orthodontically used CAD-software, but neither of them included the software used in the present investigation [30–32]. Nevertheless, agreement of current limitations in gingival boundary recognition exists and manual adjustments of tooth segmentations are deemed mandatory [18, 30, 32].

The present in vitro setup has some limitations: First, the lack of saliva, patient movements and forces generated by tongue, lips or cheeks limits transferability of the results to a certain extent. As the aforementioned factors are discussed to impede the quality of in vivo IOS [33–36], the present results could overestimate the ability of IOS to correctly display IAs in PCDs. According to the literature, the scanning software, scanning protocol, oral environment, operator experience, and scanner tip position are identified as potential sources of error [33, 36]. To minimize these variables, scanner software had been updated to the most recent version prior to the study and no further updates were performed during data acquisition. Moreover, all scans were performed by one single experienced operator using a predefined scanning protocol that specified the scan path, working distance, and a smooth, continuous movement of the scanner tip. Despite the fact that some IOS manufacturers recommend slightly altered scanning protocols [37], the scanning path occlusal – oral – buccal offers morphological landmarks, such as cusps and fissures, that serve as stable reference points for the stitching algorithm, improving matching reliability and is well established in literature [20, 38–40]. The scanning distance of 10 ± 5 mm was determined according to clinical space conditions and laid within the range of manufacturers recommendations. Nevertheless, a slight disadvantage of systems basing on active triangulation technique could have occurred, as this principle is more sensitive to alterations in scanning distance. Second, due to the in vitro character of the present investigation, the CVIs were not disinfected prior to casting, as it would be mandatory during clinical use to avoid a contamination transmission from the dental chair to the laboratory. Despite current PVS materials exhibit no significant dimensional changes during disinfection [41–44], an influence on impression accuracy by disinfection cannot be ruled out. This should be kept in mind while interpreting the achieved results of CVI impressions within the present in vitro setting. Third, the CVIs were indirectly digitized using a high-precision laboratory scanner [39, 45]. The additional step of casting gypsum models and their subsequent scanning could have caused an underestimation of IAs in the CVI group. Direct digitization of the CVIs may have reduced this potential error. However, the production of gypsum models reflects the traditional analog workflow still commonly used in clinical dentistry. Fourth, the present results have to be interpreted cautiously with regard to overall impression accuracy in terms of trueness and precision, because no reference dataset of the A-PB frasaco model was obtained. Moreover, the present investigation focused on specific IA capturing rather than full-arch accuracy as well as the data modification by software manipulation during the further processing steps and therefore used established measurements to enhance comparability with literature [14, 15]. Fifth, the measurement of IAs revealed many values of minimum and maximum IA display. Therefore, one could argue if the Median test would be most appropriate statistical analysis, because even large graphically visible differences could not be depicted by the statistical test. Nevertheless, using a generalized linear model with a logit link and a binominal regression [46, 47] instead was discussed with our professional medical statistician, but due to the large number of interactions and the much more severe interpretability of the latter, the median test was preferred. Lastly, this in vitro study was performed as single-center study. Further investigations should preferably be performed as multi-center in vivo studies to verify the obtained results.

Overall, despite the increasing prevalence of periodontitis [6], data on IOS performance in PCDs remain limited, with implications not only for orthodontics but also for prosthetic dentistry. A recent in vitro study comparing the accuracy of a single IOS with conventional impressions in partially edentulous arches with mobile teeth reported superior results for digital impressions, particularly in teeth exhibiting greater mobility [48]. The influence of interdental spacing on IOS accuracy was investigated examining veneer preparations [49–51] and with artificially created interdental spaces [52, 53]. For assessment of interproximal IOS accuracy in veneer preparations, IAs were assessed as no error, partial error (with subgroups) or total error. Different spacing thresholds ranging from 0.2 to 0.4 mm for different IOS systems were obtained [50, 51] and less errors were observed after scan postprocessing [51]. As far as comparable, the results are in line with the present investigation - while the percentage measurement of IA display of the present study may be considered advantageous for more precise assessments, the scattering of closed (0%) and open (100%) IAs revealed that a certain threshold of point distances is mandatory for current IOS systems. Artificially created interdental spacing in the posterior maxilla required distances of 0.5 to 0.6 mm under dry conditions and 0.6 to 0.7 mm under wet conditions for reliable capture by all tested IOS systems [52], which underlines the importance of further in vivo studies investigating IOS performance in PCDs. In the anterior maxilla, spaces of 1–2 mm led to higher IOS trueness compared to 0–3 mm, however, the differences were found to be clinically irrelevant [53].

## Conclusions

Within the limitations of this in vitro study, it can be concluded that IOS provide superior accuracy in capturing and reproducing IAs in PCDs compared to conventional impressions. Accuracy, however, varied between different IOS systems, highlighting the importance of selecting high-performance devices. For subsequent digital processing, including semi-automated tooth segmentation, the quality of the initial scan data is crucial. Segmentation accuracy with OnyxCep^3TM^ was higher in teeth with healthy gingival conditions, indicating that periodontal defects remain a challenge for automated CAD workflows.

## Data Availability

The data that support the findings of this study are not openly available but are available from the corresponding author upon reasonable request.
